# Low Glycolysis Is Neuroprotective during Anoxic Spreading Depolarization (SD) and Reoxygenation in Locusts

**DOI:** 10.1523/ENEURO.0325-23.2023

**Published:** 2023-11-23

**Authors:** Yuyang Wang (王宇扬), Alexander G. Little, Maria J. Aristizabal, R. Meldrum Robertson

**Affiliations:** Department of Biology, Queen’s University, Kingston, Ontario K7L 3N6, Canada

**Keywords:** anoxia-reoxygenation, CNS metabolism, glycolysis, locust, neuroenergetics, spreading depolarization

## Abstract

Migratory locusts enter a reversible hypometabolic coma to survive environmental anoxia, wherein the cessation of CNS activity is driven by spreading depolarization (SD). While glycolysis is recognized as a crucial anaerobic energy source contributing to animal anoxia tolerance, its influence on the anoxic SD trajectory and recovery outcomes remains poorly understood. We investigated the effects of varying glycolytic capacity on adult female locust anoxic SD parameters, using glucose or the glycolytic inhibitors 2-deoxy-d-glucose (2DG) or monosodium iodoacetate (MIA). Surprisingly, 2DG treatment shared similarities with glucose yet had opposite effects compared with MIA. Specifically, although SD onset was not affected, both glucose and 2DG expedited the recovery of CNS electrical activity during reoxygenation, whereas MIA delayed it. Additionally, glucose and MIA, but not 2DG, increased tissue damage and neural cell death following anoxia-reoxygenation. Notably, glucose-induced injuries were associated with heightened CO_2_ output during the early phase of reoxygenation. Conversely, 2DG resulted in a bimodal response, initially dampening CO_2_ output and gradually increasing it throughout the recovery period. Given the discrepancies between effects of 2DG and MIA, the current results require cautious interpretations. Nonetheless, our findings present evidence that glycolysis is not a critical metabolic component in either anoxic SD onset or recovery and that heightened glycolysis during reoxygenation may exacerbate CNS injuries. Furthermore, we suggest that locust anoxic recovery is not solely dependent on energy availability, and the regulation of metabolic flux during early reoxygenation may constitute a strategy to mitigate damage.

## Significance Statement

The CNS in insects can reversibly shutdown under extreme conditions like anoxia, through a process known as spreading depolarization (SD). Despite the central importance of glycolysis in CNS functioning, its precise involvement during anoxic SD remains poorly understood. Using the locust (*Locusta migratoria*) SD model, we show that glycolysis is not a critical energy source for the CNS to recover from anoxic SD, and that it could exacerbate anoxic injuries during reoxygenation. These findings identify the CNS glycolytic pathways as a potential target to mitigate detrimental effects of anoxia.

## Introduction

CNSs with restricted extracellular space and protected by diffusion barriers can reversibly shut-down because of a process known as spreading depolarization (SD; Robertson et al., 2020). In migratory locusts (*Locusta migratoria*), environmental anoxia triggers SD through the failure of aerobic metabolism and ion homeostasis, which suppresses neural activity at the onset of a hypometabolic coma (Robertson et al., 2020; Robertson and Van Dusen, 2021). SD is characterized by the abrupt collapse of ion gradients across neural cell membranes that propagates as self-regenerating waves. Such a process involves large surges of extracellular potassium ([K^+^]_o_), intracellular sodium and calcium ([Na^+^]_I_, [Ca^2+^]_i_), respectively, across the neural cell membrane, as well as the ensuing cell swelling from water influx (Robertson et al., 2020; Lemale et al., 2022). The loss of potential energy stored as trans-membrane ion gradients presents a substantial energetic challenge for SD recovery, because of the need to restore changes in the intracellular and extracellular milieu. Thus, while often fully recoverable and benign, SD frequently predisposes neural tissues to injury and cell death. At least in higher animals, including humans, SD is the characteristic change in the state of neurons that virtually always occurs in the dying process, i.e., at the transition from life to death (Carlson et al., 2018; Dreier et al., 2018, 2019). In the case of anoxic SD, although reperfusion is necessary for survival and recovery, the process of reactivating oxidative metabolism could further compound tissue injuries (Gomez et al., 2023).

Glucose availability is a critical factor modifying tissue susceptibility for SD and the resultant damage (Hoffmann et al., 2013; Sarrafzadeh et al., 2013; Lourenço et al., 2017; Rogers et al., 2017). In locusts, glycogen is depleted in the CNS following anoxia, suggesting heightened glucose utilization during anoxic SD (Hochachka, 1993). Anaerobic glycolysis is often erroneously described as the source of lowered cellular pH during SD via “lactic acid” production (Rossi et al., 2007; Uzdensky, 2019; Bo et al., 2020; Tóth et al., 2020; Grech et al., 2021; Lima et al., 2022); however, only lactate is formed which does not contribute to acidification (Robergs et al., 2018). Nonetheless, few studies have examined the impact of glycolysis and glycolytically-derived energy on SD in metabolically challenged tissues, and the conclusions have been difficult to reconcile. Some studies, for instance, show that hyperglycemia and anaerobic glycolysis enhance tissue SD resistance and reduce injury under cerebral ischemia (Hartings et al., 2008; DiNuzzo et al., 2012; Hoffmann et al., 2013; Hertz et al., 2015; Wotton et al., 2020), while others associate excess glucose supply and neuronal glycolysis with worsened secondary damage post-SD (De Courten-Myers et al., 1994; Herrero-Mendez et al., 2009; Li et al., 2019; Barros et al., 2021). In mammals, the relation between ischemic injuries and serum glucose presumably presents a U-shaped curve (Dreier and Reiffurth, 2015), and a recent matched cohort study puts the optimal serum glucose for aneurysmal subarachnoid hemorrhage (aSAH) recovery at higher than physiological level (Eagles et al., 2022).Ultimately, it remains unclear whether and how increased CNS glycolytic flux is beneficial for anoxic SD recovery or neural cell survival.

The insect CNS provides an excellent model for examining the fundamental impact of neuroenergetics on SD events. Such an approach is advantageous by circumventing the additional complexity arising from mammalian neurovascular responses (Spong et al., 2017). Both the *Drosophila* brain and the locust thoracic ganglia generate SD reliably, and share similar triggers and electrophysiological characteristics with mammalian models (Spong et al., 2016; Shuttleworth et al., 2020). Moreover, many aspects of CNS homeostasis and energy metabolism, including K^+^ spatial buffering and neuron-glia metabolite shuttling, are conserved (Spong and Robertson, 2013; Weiler et al., 2017; Rittschof and Schirmeier, 2018). Lastly, the universal reliance on glycolysis by both insect and mammalian glia underscores the central role of glycolytic flux in modulating CNS homeostasis across species (Bélanger et al., 2011; Volkenhoff et al., 2015).

In this study, we investigated the impact of CNS glycolysis on anoxic SD in the locust metathoracic ganglion (MTG). To do so, we manipulated the animal’s glycolytic capacity pharmacologically, by supplying glucose as substrate to promote glycolysis, and monosodium iodoacetate (MIA) or 2-deoxy-d-glucose (2DG) to inhibit it. We took multiple approaches to monitor the physiological effects of our manipulations on N_2_-induced anoxic SD, including electrophysiology to monitor SD trajectories and CNS ion disturbances by measuring the transperineurial potential (TPP) across the ganglion sheath, respirometry to assess the effects on aerobic metabolism, and quantifying CNS injury through neural cell death and oxidative damage.

## Materials and Methods

### Animals

Adult *L. migratoria* aged four to five weeks past adult moult were reared in a crowded colony located in the Animal Care facility at the Bioscience Complex at Queen’s University, Kingston, Ontario, Canada. The colony is maintained at around 30°C under light, and room temperature when dark, with a 12/12 h light/dark cycle. The animals were fed daily with a diet of wheatgrass, bran, milk protein, and yeast. All animals selected for experiments were aged on average five weeks past the final moult and were randomly assigned to treatment groups. Our preliminary electrophysiology experiments revealed greater influence of glucose in females compared with males. Thus, all subsequent data were collected using only females to maximize treatment effects and minimize variabilities because of sex. Animals were transported in well-ventilated plastic containers to the labs.

### Pharmacology

The final concentrations of the drugs used in all experiments were: 10 mm (180 mg/dl) for glucose, 5 mm (104 mg/dl) for MIA, and 50 mm (820 mg/dl) for 2-deoxy-d-glucose (2DG). The concentration of glucose was chosen based on a prior study, where 10 mm maximally stimulated O_2_ uptake of isolated ganglia (Clement and Strang, 1978). The reference range for hemolymph glucose concentrations is between 10 and 20 mm from start to 1 h after feeding (180–360 mg/dl; Zanotto et al., 1996). Compared with hemolymph, the semi-intact preparation allows for greater fluid volume (∼2 ml), which provides as a larger glucose reserve for the CNS. Initially, equimolar concentrations of 2DG and MIA were used with glucose. However, the concentration of 2DG was increased to 50 mm as no noticeable effects were observed (preliminary observations), and the concentration of MIA was reduced to 5 mm because of spontaneous and irreversible negative shift of TPPs.

For bath application during electrophysiology, aliquots of 100 mm glucose or 25 mm MIA or 100 mm 2DG (MIA; Sigma-Aldrich) in standard locust saline (147 mm NaCl, 10 mm KCl, 4 mm CaCl_2_, 3 mm NaOH, and 10 mm HEPES buffer; pH 7.2) were diluted to 10, 5, or 50 mm, respectively, before each experiment. For whole-animal injections, the volume of hemolymph in an animal is estimated to be 200 μl based on a prior study (Ayali and Pener, 1992). Accordingly, 30 μl of 80 mm glucose or 40 mm MIA or 400 mm 2DG in standard locust saline were delivered to achieve the appropriate final concentration in circulation.

### Electrophysiology

#### Dissection

**Figure 1.**
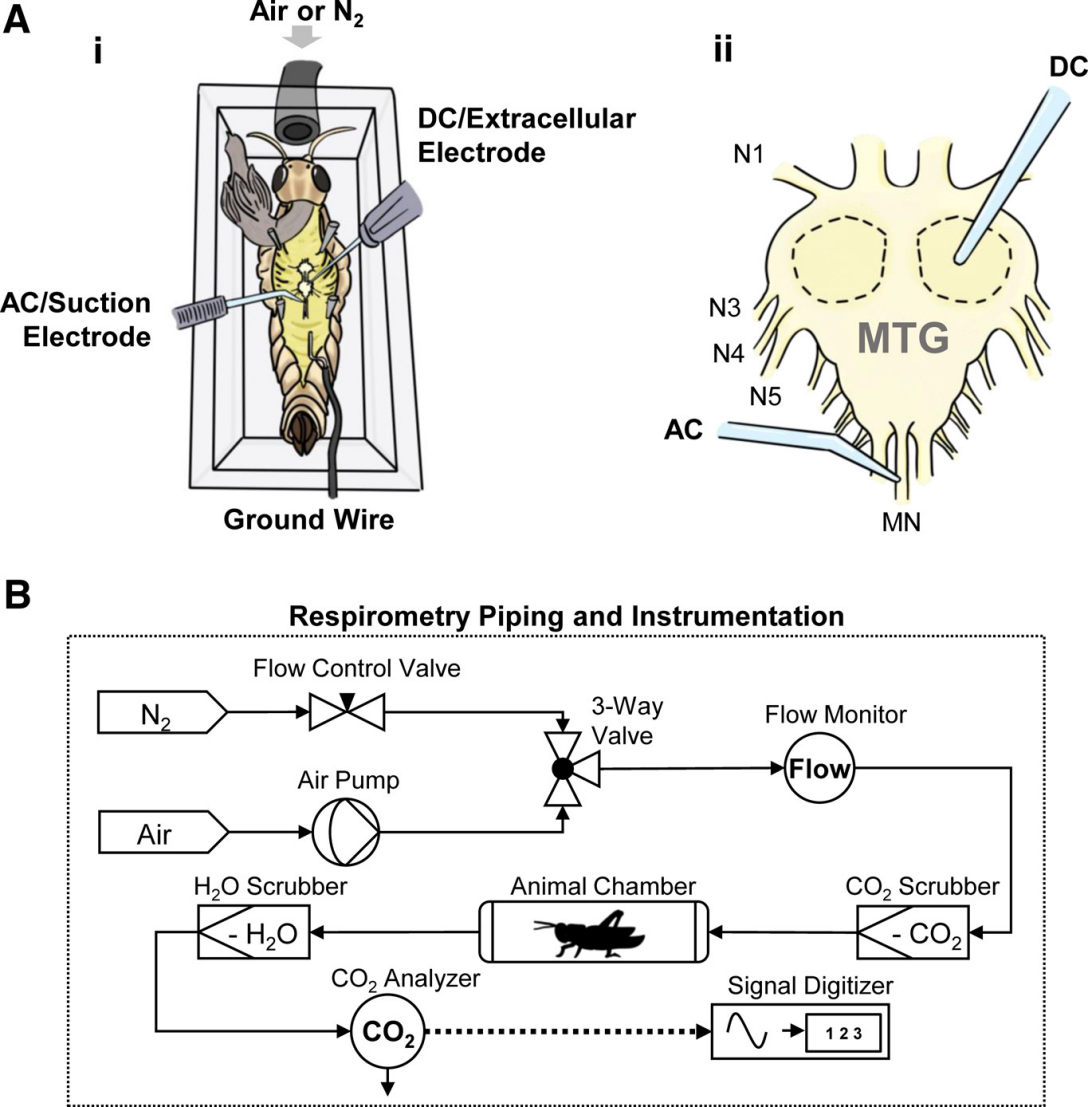
Experimental setup. ***Ai***, Diagram of semi-intact animal setup for electrophysiology, showing chamber and electrode arrangements. ***Aii***, Enlarged dorsal view of the metathoracic ganglion with electrode placements. DC electrode measures transperineurial potential (TPP) within dashed areas, while AC (alternating current) suction electrode measures ventilatory nerve rhythm on the median nerve (MN). Peripheral nerves (N1, N3, N4, and N5) serve as guiding features. ***B***, Not to scale. Schematic of flow-through respirometry system. Solid arrows indicate gas-tight vinyl tubing connections and gas flow direction; dashed arrow represents electrical connection for data acquisition. Refer to Materials and Methods for details.

An illustration of a semi-intact preparation is shown in Figure 1*Ai*. The appendages were snipped near the body and a portion of the dorsal pronotum was removed. An incision was made along the dorsal midline, starting near the seventh abdominal segment and ending at the head capsule. The animal was then pinned to the cork floor of a sealable acrylic chamber (5 × 5.2 × 2 cm), exposing the thoracic cavity. The air sacs, fat bodies and eggs were subsequently removed, and the gut was clipped at the posterior end and pulled over the head capsule. Standard locust saline was added into the thoracic cavity immediately afterward to prevent dehydration. The ventral diaphragm was then removed, exposing the metathoracic ganglion and the associated nerve roots. An aquarium air pump ventilated the chamber during dissection and subsequent recording sessions with a flow rate of 100 ml/min.

#### Recording setup

The animal was grounded using a chlorided silver wire placed at the caudal end of the abdominal cavity in contact with bathing saline. The electrode placements are shown in Figure 1*Aii*. TPP was measured with an extracellular DC electrode made from 1-mm filamented glass capillary (World Precision Instruments) and pulled to a tip resistance of ∼5 MΩ. The electrode and holder were filled with 500 mm and 3 m KCl, respectively, and connected to a DC amplifier (Model 1600, A-M Systems). The DC electrode was calibrated in the bathing saline before penetration of MTG at the start of the experiment and after the removal from the MTG at the end of the experiment, to adjust for baseline shift. Nerve activity was measured by an AC suction electrode on the dorsal median nerve of the MTG. The AC electrode was made from 1-mm nonfilamented glass capillary (World Precision Instruments) and pulled to ∼5-MΩ tip resistance; the tip was then broken off with forceps to align with the diameter of the dorsal median nerve. The AC electrode was connected to a differential AC amplifier (model 1700, A-M Systems). The analog signals from both amplifiers were digitized (DigiData 1322A, Molecular Devices) and recorded with Axoscope 10.7 (Molecular Devices).

#### Recordings of anoxic SD

Each day of the experiment included an equal number of control and treatment animals to account for daily variations in the colony. Following dissection, the saline in the thoracic cavity was changed to either the treatment or the control saline (washed three times), with a 20-min pretreatment period before anoxia. During pretreatment, the DC and AC electrodes were calibrated and positioned in place, and the chamber was sealed with cellophane tape. N_2_ anoxia was introduced immediately following the pretreatment period; 10 min after the abrupt negative DC shift marking SD onset, the N_2_ was switched off and the air pump circulated the air back to reoxygenate the animal. A constant flowrate of 100 ml/min was maintained for air and N_2_.

The time to motor pattern failure (F_patt_), the time to excitability failure (F_exc_) and the time to SD (F_TPP_) were the times taken from the start of anoxia to the disappearance of distinct rhythmical nerve activity, nerve excitation, and the abrupt negative DC shift of TPP, respectively. The time to motor pattern recovery (R_patt_), the time to excitability recovery (R_exc_) and the time to SD recovery (R_TPP_) were the times taken for the return of rhythmical nerve activity, nerve excitation, and the positive peak value of TPP after the air returns, respectively. SD amplitude was taken as the difference between the baseline TPP preanoxia and the steady TPP value immediately following the abrupt negative shift; post-SD positivity represents the difference between the maximum recovered TPP value and the preanoxia baseline. TPP recovery slope was measured as the linear slope of TPP increase at the initial phase of recovery. The recovery time constant (τ) was obtained by fitting the DC recovery trace with the standard exponential model in Clampfit 10.7 (Molecular Devices).

### Molecular and biochemical assays

#### Tissue preparation

Intact animals were restrained with scotch tape and injected with either saline or drugs using a 50-μl Hamilton syringe (Hamilton Company). They were then placed in the same chamber used in respirometry experiments for 30 min following injection for pretreatment, and subjected to 30-min N_2_ anoxia except for control animals, which did not receive anoxia. The animals subsequently recovered for 2 h in normoxia to allow injuries to develop, before each MTG was extracted through a ventral incision and snap frozen in liquid N_2_ for later use. All tissue samples were homogenized in lysis buffers supplied by the corresponding assay kits, using a TissueLyzer II bead mill (one 5-mm stainless steel bead per tube, 30 s^−1^ frequency for 1.5 min; QIAGEN Inc.). The homogenate protein concentrations were determined using a QuantiPro BCA protein assay kit (Sigma-Aldrich; catalog #QPBCA). All colorimetric and fluorometric assays were performed using the SpectraMax Paradigm microplate reader (Molecular Devices).

#### Caspase-3 activity assay

The level of neural caspase three activation was assessed using a Caspase-3 Assay kit, Fluorometric (Sigma-Aldrich; catalog #CASP3F), following manufacturer’s instructions. Modifications were made to recommended kit reagent volumes to ensure each sample assayed an individual MTG. In brief, each MTG was homogenized in 150-μl lysis buffer, loaded as duplicates on a 96-well microplate (50 μl per well), and then reacted with equal volume of 2× substrate working solutions. The concentration of fluorescent end-product 7-amino-4-methylcoumarin (AMC) was measured with excitation and emission wavelength of 360 and 460 nm, respectively. Results are presented as μmol AMC cleavage per hour per mg protein in the sample. Outliers in data were removed using Grubb’s test online (https://www.graphpad.com/quickcalcs/grubbs1/), figure with outliers is presented in Extended Data Figure 7-1.

#### TBARS assay

The extent of lipid peroxidation damage was estimated with QuantiChrom TBARS Assay kit (BioAssay Systems; catalog #DTBA-100), following manufacturer’s instructions. Briefly, two MTGs were pooled into each sample, homogenized in 200 μl of cold PBS. The homogenate was then incubated with equal volumes of thiobarbituric (TBA) reagent for 1 h at 100°C. The reaction mixtures were cooled and loaded into a 96-well microplate in duplicates, then measured fluorometrically using excitation and emission wavelength of 530 and 550 nm, respectively. Results are presented as μmol MDA per mg protein.

#### Immunoblotting

The protein carbonyl content in ganglionic tissue was measured with Protein Carbonyl Assay kit (Western blotting; Abcam; catalog #ab178020), following manufacturer’s instructions. In short, two MTGs were pooled in each sample and homogenized in 100 μl tris-buffered saline (pH 7.5) containing cOmplete ULTRA protease inhibitor cocktail (Sigma-Aldrich). The homogenates were combined with equal volumes of 2× extraction buffer and spun at 18,000 × *g* for 20 min at 4°C, the supernatants were split equally and reacted with either dinitrophenylhydrazine (DNPH) or control solutions before gel electrophoresis.

To optimize protein loading amounts, a standard curve was generated and analyzed using the Empiria 2.0 software linear range detection function (LI-COR Biosciences). 20 μg of control or derivatized protein were resolved on a 7.5% SDS-PAGE gel and transferred onto a nitrocellulose membrane (0.45 μm pore size). Total protein signal was measured with the Revert 700 total protein stain kit (LI-COR), before acquiring the signal for anti-dinitrophenyl (DNP; supplied in ab178020, 1:5000). An Odyssey XF imaging system was used to image the membrane; the whole-lane signals were quantified and then normalized against total protein, using the Empiria 2.0 software (LI-COR Biosciences). Data were compiled from five blots (all biological replicates), and normalized signal intensities are plotted as fold change compared with control. The unprocessed blot images are provided in Extended Data Figure 7-2.

#### Respirometry

A simple flow-through respirometry system was constructed (Fig. 1*B*). A manually operated three-way valve controlled the feeding of either compressed N_2_ or normoxic air supplied by an air pump (Qbit Systems Inc.), which passed through a CO_2_ scrubber containing soda lime before entering the animal chamber. Water vapor was removed with a Drierite filter before the gas entered an infrared CO_2_ analyzer (QS-151, Qbit Systems Inc.); the analog signals from the analyzer were digitized (DigiData 1322A, Molecular Devices) and acquired in Axoscope 10.7 (Molecular Devices). The flowrate of oxygenated air and N_2_ were maintained at a constant 100 ml/min as indicated by the flow monitor (Q-G268, Qbit Systems Inc.).

Intact animals were used for respirometry and were injected as described above, and immediately placed in the chamber. The animals were allowed 30 min to recover from manipulation and for their CO_2_ output to stabilize under normoxic air. Afterwards, N_2_ was switched on to induce a 35-min anoxia, followed by 2 h of recovery in normoxic air. Following recovery, the MTG was immediately collected and stored to supplement downstream molecular and biochemical assays. Data analysis was performed in Clampfit 10.7 (Molecular Devices), where the CO_2_ emission trace was integrated to calculate gas output for a given time interval. Raw data in PPM were converted to emission rate expressed in μl CO_2_ per minute using the following formula:

$$CO_{2}\;Emission\;Rate\left(\mu L\;min^{-1}\right)=\left[CO_{2}\right]\left(PPM\right)\times Flow\;Rate(L\;min^{-1}).$$

### Data analysis

SigmaPlot 13 (SPSS Inc.) was used to analyze data and produce figures. α Was set at *p* = 0.05. Data normality and equality of group variances were determined by Shapiro–Wilk and Brown–Forsythe tests, respectively. For electrophysiology results, the means of normally distributed data were compared by two-samples independent *t* test; otherwise, Mann–Whitney ranked sum test was used to compare medians. For all other data, one-way ANOVA test was used. For representative traces in the figures, the sample closest to the mean were selected and processed using Clampfit 10.7 (Molecular Devices), where abrupt and short-lived (<1 ms) electrical artifacts were replaced with local mean values.

## Results

### Both glucose and 2DG promote recovery of CNS ion homeostasis

**Figure 2.**
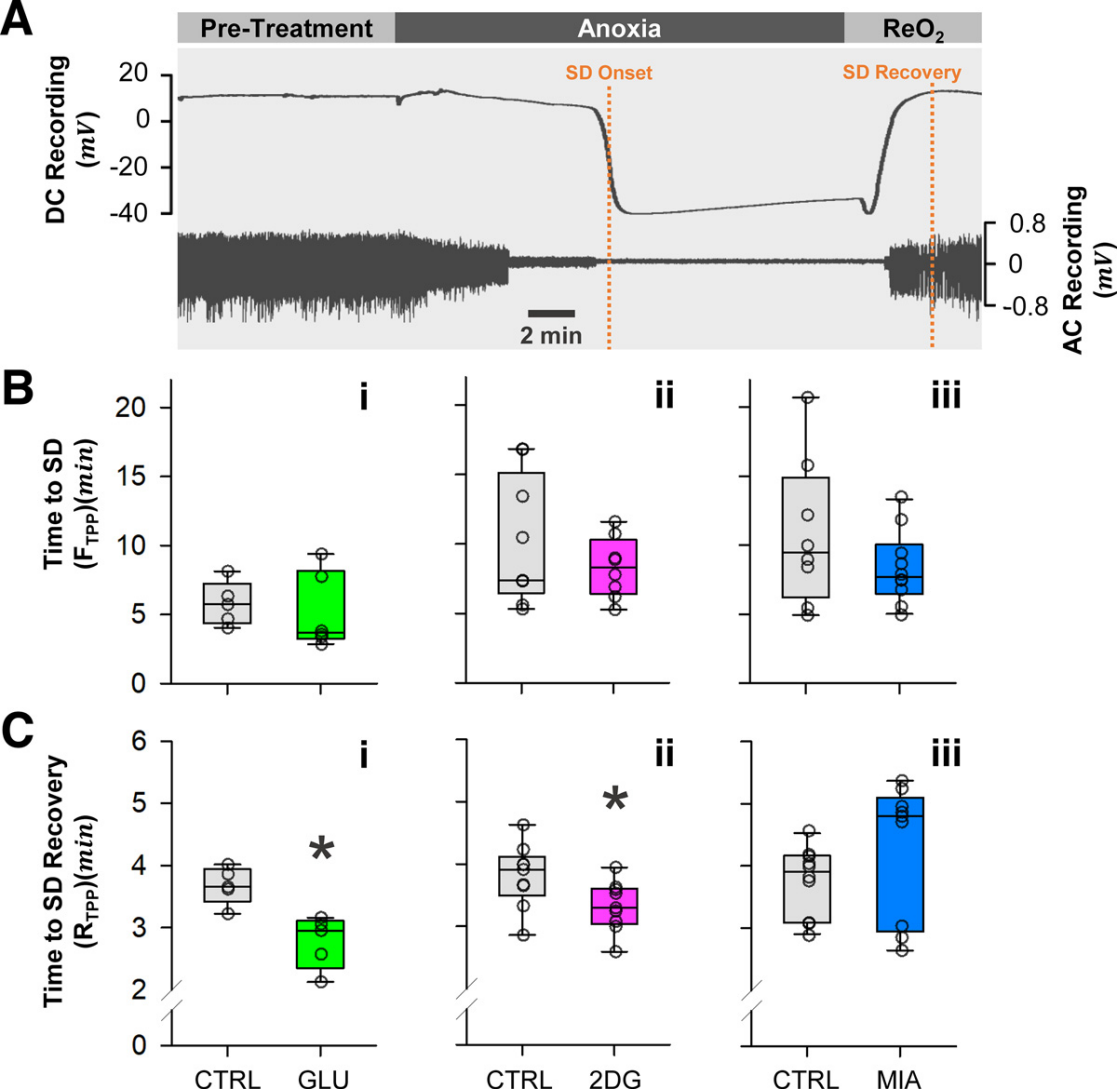
SD onset and recovery times. ***A***, Representative recording showing TPP and nerve activity. SD onset (F_TPP_) is defined as the time when TPP reaches half-maximal amplitude of the abrupt negative shift during anoxia, while SD recovery (R_TPP_) is defined as the time when TPP reaches its maximal value during reoxygenation. ReO_2_: Reoxygenation period. ***Bi***, ***Bii***, ***Biii***, Glycolytic capacity has no effect on F_TPP_. ***Ci***, ***Cii***, ***Ciii***, Glucose and 2DG significantly shorten R_TPP_, while MIA has no significant effect. Plotted data represent medians and IQR. Asterisks (*) indicate *p* < 0.05. Refer to Results for sample sizes and *p*-values.

To determine whether glycolytic capacity influences anoxic SD time course and trajectory, we measured the times to TPP failure and recovery (Fig. 2). Pretreatment with 10 mm glucose, 5 mm MIA, and 50 mm 2DG did not significantly influence the time to SD (F_TPP_) during N_2_ anoxia (two-sample Student’s *t* test, vs control; F_TPP-Glu_, Control: *n* = 5, Glucose: *n* = 6, *p* = 0.65; F_TPP-MIA_, Control: *n* = 8, MIA: *n* = 10, *p* = 0.34; F_TPP-2DG_, Control: *n* = 9, 2DG: *n* = 8, *p* = 0.22; Fig. 2*B*). However, 10 mm glucose and 50 mm 2DG significantly shortened the time to TPP recovery (R_TPP_) on reoxygenation (two-sample Student’s *t* test, vs control; R_TPP-Glu_, Control: *n* = 5, Glucose: *n* = 6, *p* = 0.032; R_TPP-2DG_, Control: *n* = 9, 2DG: *n* = 8, *p* = 0.040; Fig. 2*Ci*,*Cii*). On the other hand, 5 mm MIA had no significant effect on R_TPP_ (two-sample Student’s *t* test, vs control; R_TPP-MIA_, Control: *n* = 10, MIA: *n* = 9, *p* = 0.11; Fig. 2*Cii*).

**Figure 3.**
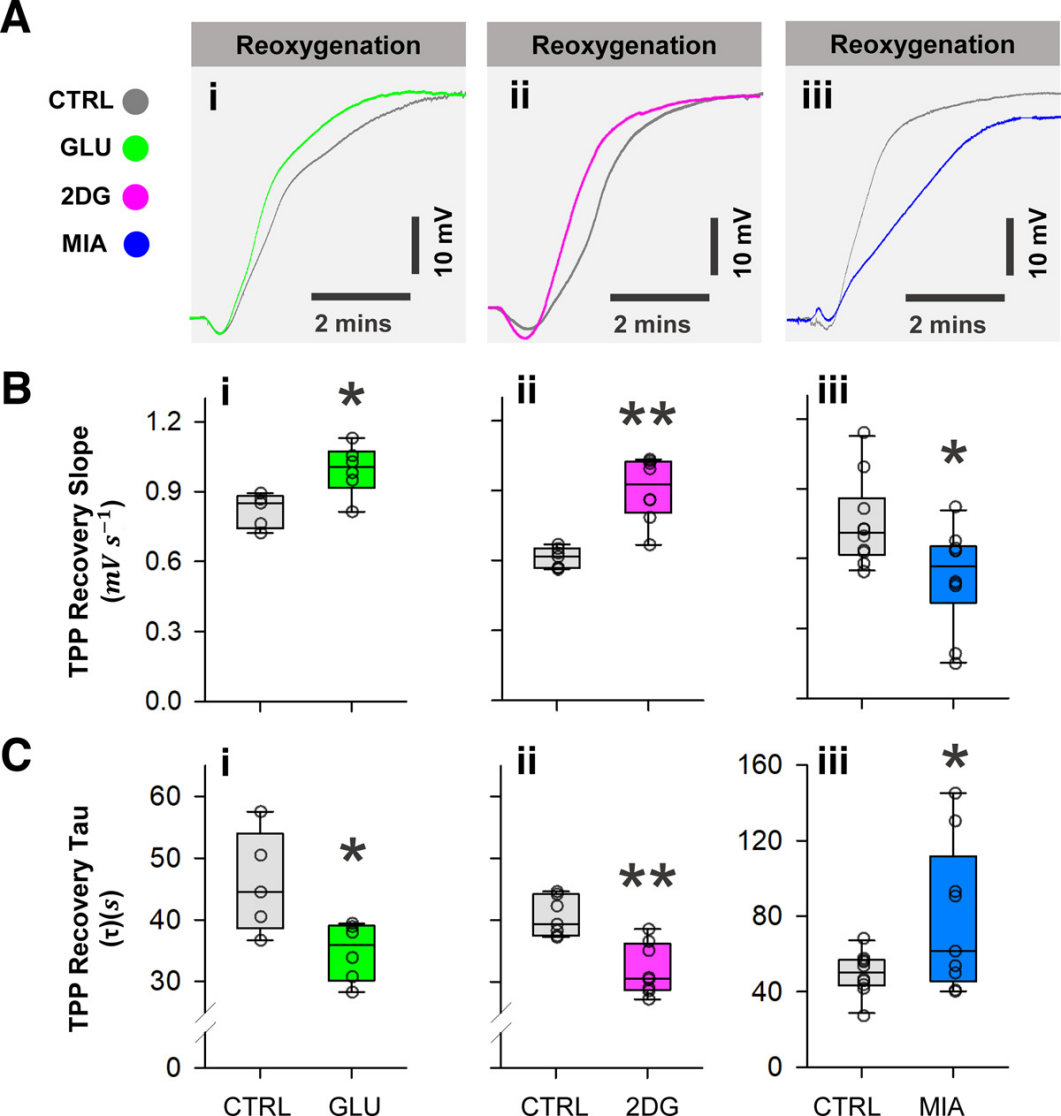
SD recovery slopes and time constants τ. ***Ai***, ***Ai***, ***Aiii***, Representative recordings overlay showing TPP recovery trajectory in glucose, 2DG, and MIA groups, respectively, starting at the onset of reoxygenation. ***Bi***, ***Bii***, ***Biii***, Glucose and 2DG increase the initial slope of TPP recovery, while MIA decreases the slope. ***Ci***, ***Cii***, ***Ciii***, Corresponding to the changes in recovery slope, glucose, and 2DG decrease the time constants (τ) of the exponential recovery trajectory, whereas MIA increases τ. Note the different scale in ***Ciii*** for clarity. Plotted data represent medians and IQR. Single asterisks (*) indicate *p* < 0.05, double asterisks indicate *p* < 0.001. Refer to Results for sample sizes and *p*-values.

To compare recovery kinetics between different pretreatment regimens, we calculated the initial linear phase TPP recovery rate and the recovery time constant τ (Fig. 3). 10 mm glucose pretreatment had a significantly higher initial rate of TPP recovery (two-sample Student’s *t* test, vs control; Control: *n* = 5, Glucose: *n* = 6, *p* = 0.042; Fig. 3*Ai*,*Bi*); similarly, 50 mm 2DG also increased the initial recovery rate (Mann–Whitney ranked sum test, vs control; Control: *n* = 7, 2DG: *n* = 8, *p* < 0.001; Fig. 3*Aii*,*Bii*). The increased initial rates in glucose and 2DG groups are associated with reduced τ (two-sample Student’s *t* test, vs control; τ _Glu_, Control: *n* = 5, Glucose: *n* = 6, *p* = 0.020; τ _2DG_, Control: *n* = 7, 2DG: *n* = 8, *p* < 0.001; Fig. 3*Ci*,*Cii*). Despite having no significant effect on R_TPP_, 5 mm MIA reduced the initial recovery rate and lengthened τ (two-sample Student’s *t* test, vs control; initial rate, Control: *n* = 10, MIA: *n* = 10, *p* = 0.022; τ _MIA_, Control: *n* = 10, MIA: *n* = 10, *p* = 0.039; Fig. 3*Aiii*,*Biii*,*Ciii*).

### Excitability recovery is hastened by glucose and 2DG while delayed by MIA

**Figure 4.**
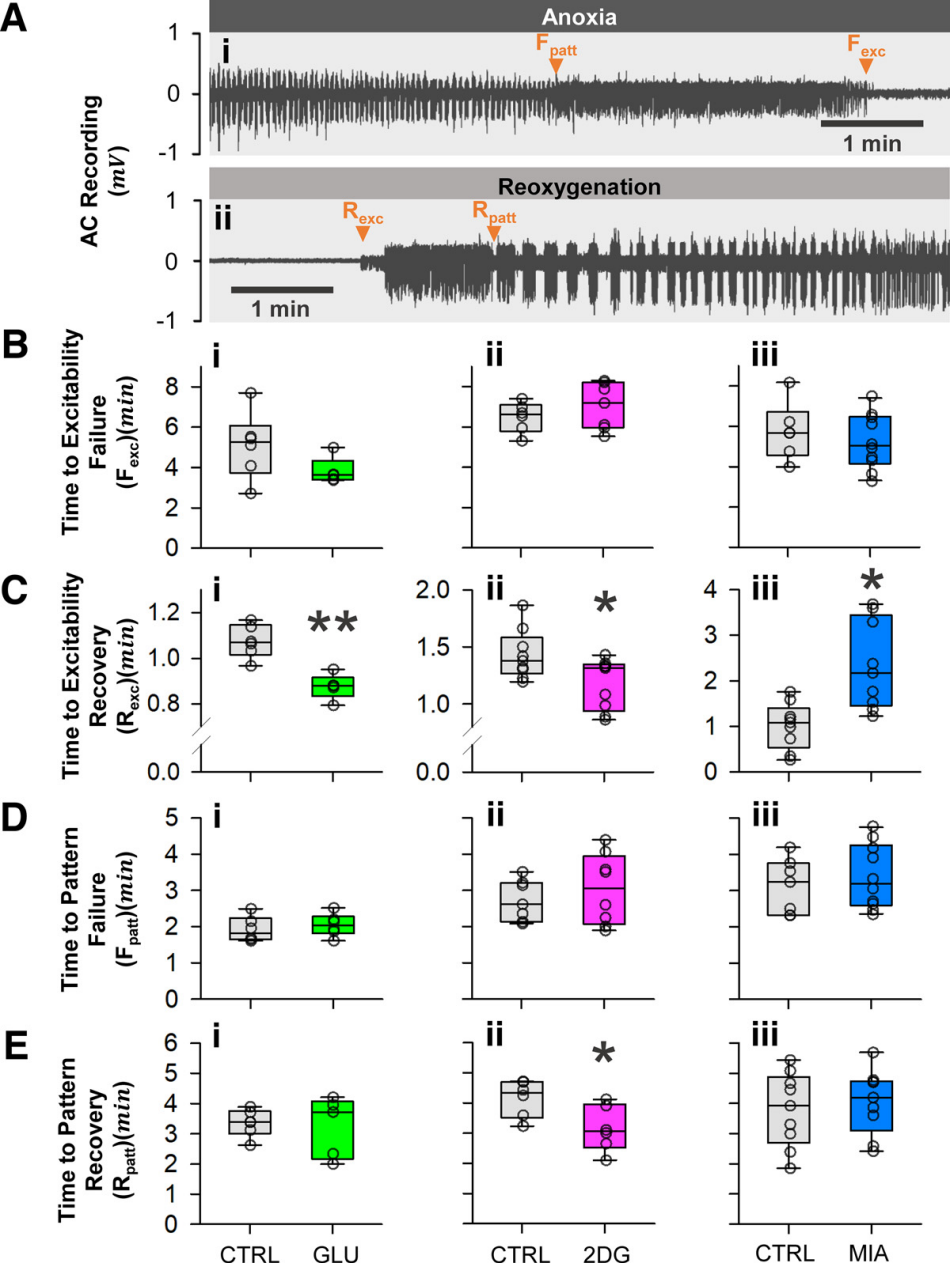
Excitability and motorpattern failure and recovery times. ***Ai***, ***Aii***, Representative recordings of nerve activity during anoxia and reoxygenation, respectively, showing excitability failure (F_exc_) and recovery (R_exc_) defined as the disappearance and re-emergence of randomized spikes, and motor pattern failure (F_patt_) and recovery (R_patt_) defined as the disappearance and re-emergence of synchronized action potentials. ***Aii***, Trace starts at the onset of reoxygenation. ***Bi***, ***Bii***, ***Biii***, None of the treatments affect F_exc_. ***Ci***, ***Cii***, ***Ciii***, Glucose and 2DG hasten R_exc_, while MIA delays the recovery. Note the different scales for clarity. ***Di***, ***Dii***, ***Diii***, None of the treatments affect F_patt_. ***Ei***, ***Eii***, ***Eiii***, Only 2DG accelerates R_patt_, while glucose and MIA have no effect. Plotted data represent medians and IQR. Single asterisks (*) indicate *p* < 0.05, double asterisks indicate *p* < 0.001. Refer to Results for sample sizes and *p*-values.

To examine whether glycolytic capacity affect action potential generation, we measured the time to neuronal excitability failure and recovery (Fig. 4*A*). Neither 10 mm glucose nor 5 mm MIA affected time to excitability failure (F_exc_) under N_2_ anoxia (two-sample Student’s *t* test, vs control; F_exc-Glu_, Control: *n* = 6, Glucose: *n* = 5, *p* = 0.14; F_exc-MIA_, Control: *n* = 6, MIA: *n* = 10, *p* = 0.49; Fig. 4*Bi*,*Bii*). Similarly, 50 mm 2DG did not influence F_exc_ (Mann–Whitney ranked sum test, vs control; F_exc-2DG_, Control: *n* = 6, 2DG: *n* = 7, *p* = 0.37; Fig. 4*Bii*). On the other hand, the pretreatment regimens had differential effects on time to excitability recovery (R_exc_; Fig. 4*C*). Specifically, 10 mm glucose and 50 mm 2DG both hastened R_exc_ (two-sample Student’s *t* test, vs control; R_exc-Glu_, Control: *n* = 6, Glucose: *n* = 5, *p* < 0.001; R_exc-2DG_, Control: *n* = 9, 2DG: *n* = 9, *p* = 0.026; Fig. 4*Ci*,*Cii*), whereas 5 mm MIA delayed R_exc_ (Mann–Whitney ranked sum test, vs control; R_exc-MIA_, Control: *n* = 9, MIA: *n* = 9, *p* = 0.003; Fig. 4*Ciii*).

### Only 2DG improves motor patterning recovery

To resolve the effect of glycolytic capacity on the operation of neuronal circuits during anoxic SD, we also measured the times to ventilatory motor pattern failure and recovery (Fig. 4*A*). None of the pretreatment regimens influenced the time to motor pattern failure (F_patt_) during N_2_ anoxia (Fig. 4*D*; two-sample Student’s *t* test, vs control; F_patt-Glu_, Control: *n* = 6, Glucose: *n* = 6, *p* = 0.54; F_patt-MIA_, Control: *n* = 7, MIA: *n* = 10, *p* = 0.53; F_patt-2DG_, Control: *n* = 7, 2DG: *n* = 8, *p* = 0.47). However, 50 mm 2DG significantly shortened the recovery time of motor pattern (R_patt_) following reoxygenation (two-sample Student’s *t* test, vs control; R_patt-2DG_, Control: *n* = 6, 2DG: *n* = 6, *p* = 0.03; Fig. 4*Eii*). Neither 10 mm glucose nor 5 mm MIA affected motor pattern recovery times (two-sample Student’s *t* test, vs control; R_patt-Glu_, Control: *n* = 6, Glucose: *n* = 5, *p* = 0.81; R_patt-MIA_, Control: *n* = 9, MIA: *n* = 9, *p* = 0.63; Fig. 4*Ei*,*Eii*).

### MIA and 2DG differentially impact post-SD positivity, while glucose attenuates SD amplitude

**Figure 5.**
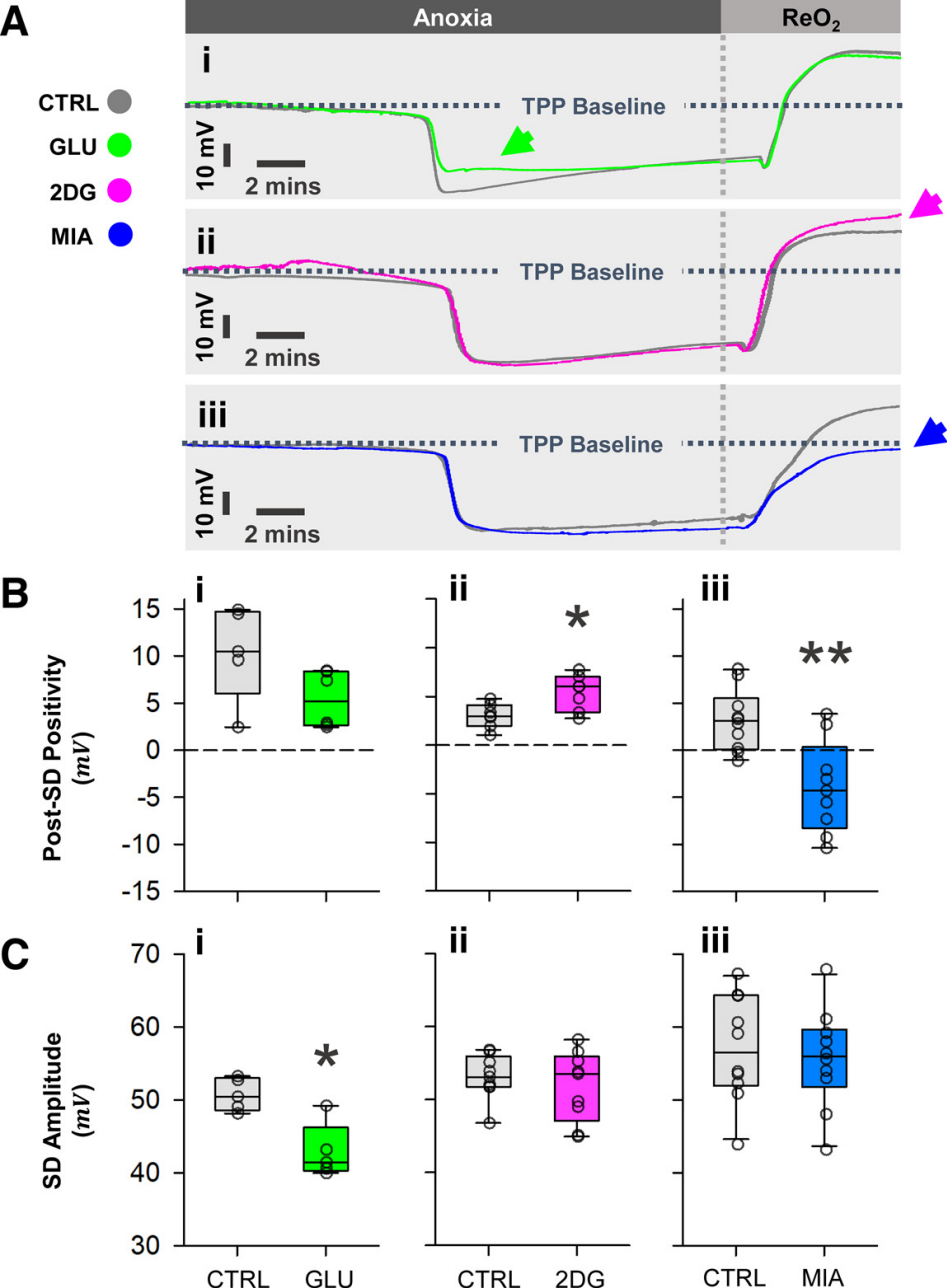
Post-SD positivity and SD amplitude. ***Ai***, ***Aii***, ***Aiii***, Representative recordings overlay showing the trajectory of TPP. Horizontal dashed line represents the baseline TPP level before anoxia, which typically persist for a period under anoxia until SD onset. Vertical dashed line indicates the onset of reoxygenation (ReO_2_). The arrow in ***Ai*** highlights attenuated SD amplitude in the glucose group, while arrows in ***Aii*** and ***Aiii*** show differences in post-SD positivity. ***Bi***, ***Bii***, ***Biii***, Glucose has no effect on DC positivity, 2DG increases its magnitude, and MIA has the opposite effect. ***Ci***, ***Cii***, ***Ciii***, Only glucose reduces SD amplitude. Plotted data represent medians and IQR. Single asterisks (*) indicate *p* < 0.05, and double asterisks indicate *p* < 0.001. Refer to Results for sample sizes and *p*-values. See Extended Data Figure 5-1 for the proposed mechanism of post-SD positivity in insects.

An unexpected observation is that TPP baseline postanoxia was differentially affected by MIA and 2DG (Fig. 5*A*,*B*). Normally, the TPP trajectory during recovery briefly surpasses the preanoxic baseline by 2–5 mV (post-SD positivity) before returning to preanoxic baseline level (Fig. 5*A*). Glucose did not affect the amplitude of DC positivity (two-sample Student’s *t* test, vs control; Control: *n* = 5, Glucose: *n* = 6, *p* = 0.071; Fig. 5*Ai*,*Bi*). However, pretreatment with 50 mm 2DG significantly increased the DC positivity (two-sample Student’s *t* test, vs control; Control: *n* = 7, 2DG: *n* = 7, *p* = 0.036; Fig. 5*Aii*,*Bii*); whereas 5 mm MIA resulted in a persistent depression of TPP compared with the preanoxic levels (two-sample Student’s *t* test, vs control; Control: *n* = 10, MIA: *n* = 9, *p* = 0.0016; Fig. 5*Aiii*,*Biii*).

10.1523/ENEURO.0325-23.2023.f5-1 Extended Data Figure 5-1 Theoretical origin of the post-SD positivity. ***A***, Illustration demonstrating the relationship between V_a_, V_b_, and TPP. ***B***, Theorized recovery dynamics of V_a_ and V_b_ and their effect on post-SD positivity. ***Bi***, Control. ***Bii***, Glucose increases the rate of both V_a_ and V_b_ recovery, having limited effects on DC positivity. ***Biii***, 2DG only increases the rate V_a_ recovery, leading to greater DC positivity. ***Biv***, Overlay of simulated TPP recovery trajectories. All traces begin at the onset of reoxygenation. Download Figure 5-1, TIF file.

To determine whether the altered postanoxic TPP level from 2DG and MIA group originates from different magnitudes of negative DC shift, we measured the SD amplitudes (Fig. 5*C*). Surprisingly, 10 mm glucose significantly reduced SD amplitudes (two-sample Student’s *t* test, vs control; Control: *n* = 5, Glucose: *n* = 6, *p* = 0.027; Fig. 5*Ai*,*Ci*). Neither 5 mm MIA nor 50 mm 2DG affected SD amplitudes (two-sample Student’s *t* test, vs control; MIA, Control: *n* = 10, MIA: *n* = 10, *p* = 0.67; 2DG, Control: *n* = 9, 2DG: *n* = 9, *p* = 0.5; Fig. 5*Aii*,*Aiii*,*Cii*,*Ciii*).

### 2DG and glucose have opposing, temporally dependent effects on CO_2_ emission during reoxygenation

**Figure 6.**
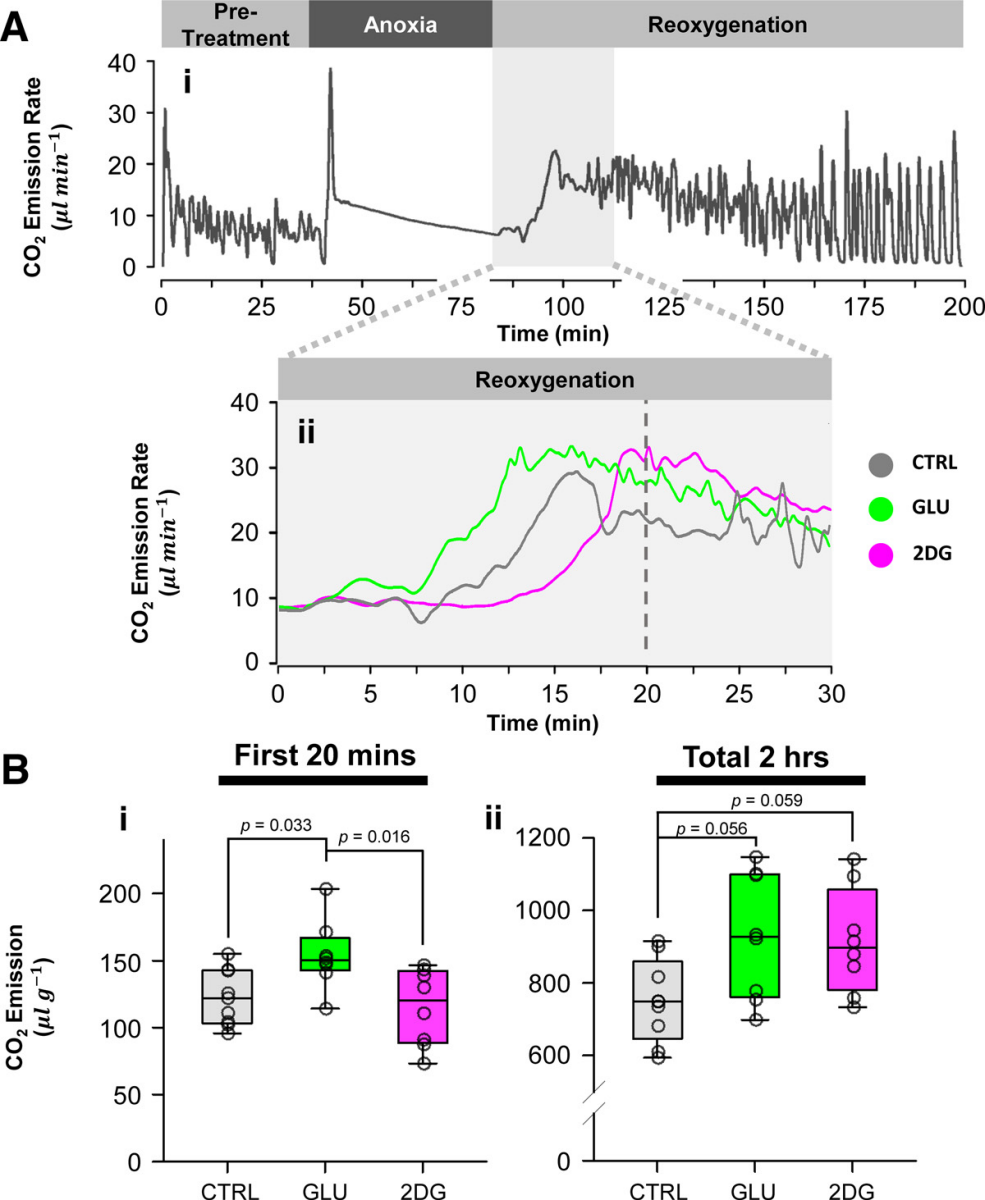
Effects on aerobic metabolism. ***Ai***, Representative CO_2_ emission trace during the respirometry experiment. ***Aii***, Overlay of representative recordings from control, glucose, and 2DG groups, showing differential CO_2_ emission patterns during the initial 30 min of reoxygenation. Glucose induces a rapid increase in emission rate, while 2DG has the opposite effect. Vertical dashed line indicates the 20 min mark, where treatment differences are most pronounced. ***Bi***, ***Bii***, CO_2_ emission per gram of body mass during the first 20 min and the total 2-h reoxygenation period, respectively. Glucose animals emit higher CO_2_ volume during the initial 20 min, while both glucose and 2DG increase CO_2_ release over the 2-h recovery period. Plotted data represent medians and IQR. Refer to Results for sample sizes.

Considering that 2DG inhibits glycolysis and thus, pyruvate production, we hypothesized that 2DG also influences aerobic metabolism. Whole animal flow-through respirometry revealed a striking observation that both control and glucose experienced a rapid rise in CO_2_ emissions during reoxygenation, whereas 2DG led to a slower and more steady increase (Fig. 6*A*). MIA results were not included as the treatments completely abolished ventilation pattern and was deemed too toxic for the animals. Interestingly, only glucose significantly increased CO_2_ output during the first 20 min of reoxygenation, the period that corresponds to the re-establishment of CNS ion-homeostasis and functioning (one-way ANOVA, Control: *n* = 9, Glucose: *n* = 8, 2DG: *n* = 8, *F* = 5.472, *p* = 0.012; Holm–Sidak pairwise comparison, glucose vs control, *p* = 0.033; glucose vs 2DG, *p* = 0.016; 2DG vs control, *p* = 0.56; Fig. 6*Bi*). On the other hand, both 2DG and glucose pretreatments significantly increased total CO_2_ emission in the 2-h period following reoxygenation (one-way ANOVA, Control: *n* = 9, Glucose: *n* = 8, 2DG: *n* = 8, *F* = 4.033, *p* = 0.032; Fig. 6*Bii*).

### The pattern of CNS damage following anoxic SD recovery

**Figure 7.**
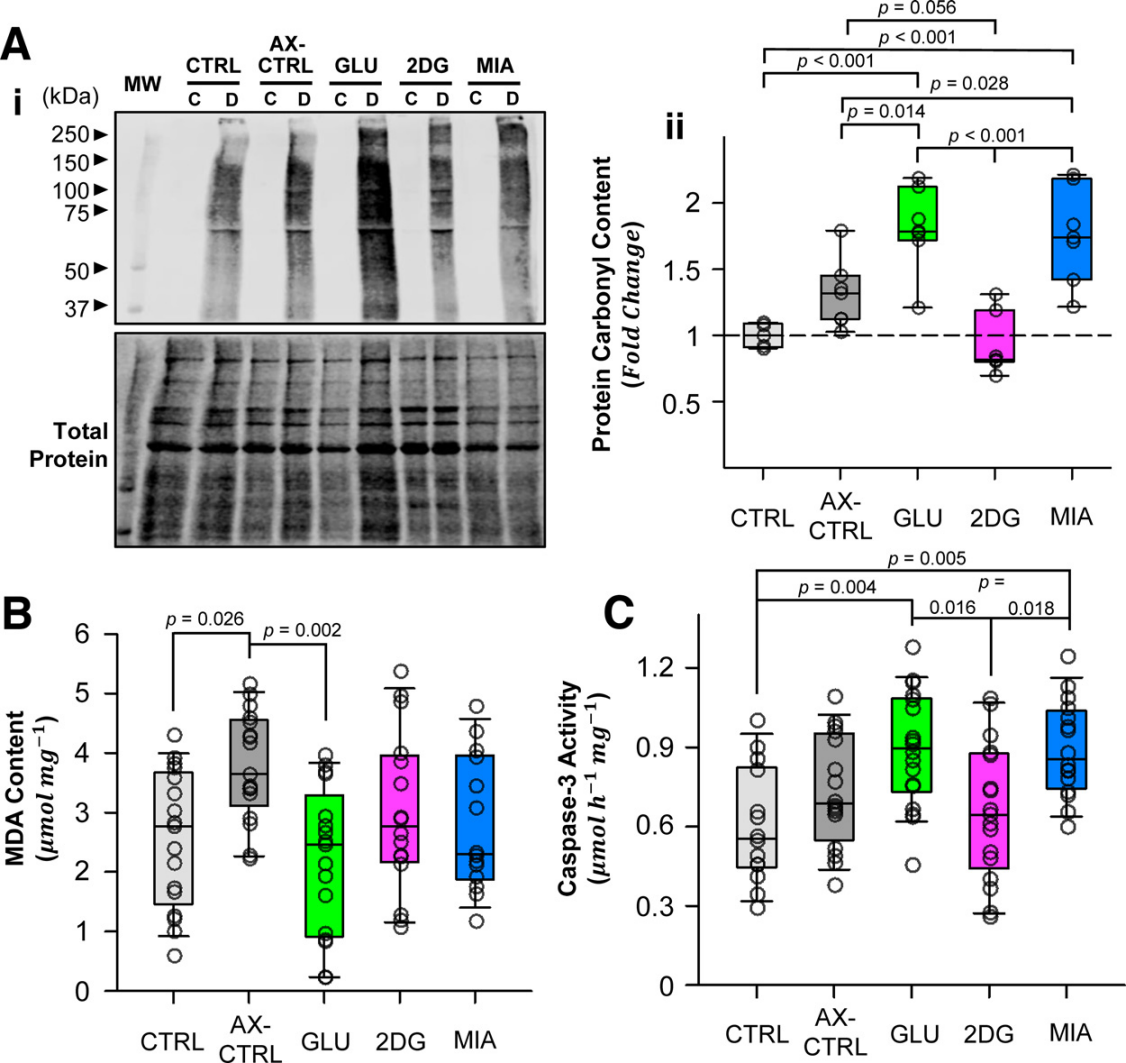
Patterns of tissue damage postanoxic SD. ***Ai***, Western blotting detecting protein carbonylation in ganglionic homogenates, with total protein stain included for reference. Each sample (2 ganglia) is loaded in two lanes: C (control, no DNPH) and D (derivatized, with DNPH). ***Aii***, Western blotting quantification shows that glucose and MIA significantly increase protein carbonylation. ***B***, Anoxia significantly increases lipid peroxidation end-product MDA per mg protein content measured in TBARS assay, while glucose reduces MDA levels similar to control. ***C***, Glucose and MIA induce greater neural apoptosis, as measured by Caspase-3 activity per mg protein. Each data point corresponds to a single animal in TBARS and Cas-3 activity assays. CTRL animals represent baseline damage levels, and AX-CTRL are anoxic control animals. Plotted data represent medians and IQR. Refer to Results for sample sizes. See Extended Data Figures 7-1 and 7-2 for Cas-3 activity assay result containing outliers and unaltered Western blotting images, respectively.

To examine whether improved electrophysiological performance corresponds to reduced CNS damage following anoxic SD, we measured the protein carbonyls levels and tissue malondialdehyde (MDA) content after N_2_ anoxia, to quantify the extent of protein oxidation and membrane lipid peroxidation, respectively (Fig. 7*A*,*B*).

10.1523/ENEURO.0325-23.2023.f7-1 Extended Data Figure 7-1 Caspase-3 activity assay data with outliers. Download Figure 7-1, TIF file.

10.1523/ENEURO.0325-23.2023.f7-2 Extended Data Figure 7-2 Unaltered blot images used in protein carbonyl detection. Subpanel ***i*** displays anti-DNP signals, subpanel ***ii*** displays corresponding total protein stain. ***A–C***, Each derivatized sample [labeled as D (derivatized) in Fig. 7] has a corresponding sham treatment [labeled as C (control) in Fig. 7]. ***D***, ***E***, All samples are combined into a single sham treatment lane. See legend for abbreviations. Download Figure 7-2, TIF file.

Protein carbonyl content significantly differed between pretreatment regimens (one-way ANOVA, Control: *n* = 5, Anoxic Control: *n* = 7, Glucose: *n* = 7, 2DG: *n* = 7, MIA: *n* = 7; *F* = 14.575; *p* < 0.001; Fig. 7*A*); anoxia alone and 2DG had no effect (Holm–Sidak multiple comparison, anoxic control vs control, *p* = 0.18; 2DG vs control, *p* = 0.88), but glucose and MIA pretreatment significantly increased protein carbonyl levels compared with both control groups and 2DG group (glucose or MIA vs control or 2DG, *p* < 0.001; glucose vs anoxic control, *p* = 0.014; MIA vs anoxic control, *p* = 0.028; Fig. 7*Aii*). An interesting trend that emerged is that 2DG pretreatment reduced protein carbonylation compared with the anoxic control (*p* = 0.056). The pretreatment regimens and anoxia significantly affected ganglionic MDA content (one-way ANOVA, Control: *n* = 17, Anoxic Control: *n* = 17, Glucose: *n* = 17, 2DG: *n* = 16, MIA: *n* = 14; *F* = 4.394; *p* = 0.003; Fig. 7*A*); anoxic control accumulated more MDA than control animals in normoxic conditions (Holm–Sidak multiple comparison; *p* = 0.026; Fig. 7*B*), glucose reduced MDA content compared with the anoxic controls (*p* = 0.002), 2DG and MIA had no effect on MDA content following anoxia (*p* = 0.32 and *p* = 0.16, respectively).

Energy stress and cellular injuries could lead to neural cell apoptosis following anoxia-reoxygenation. We assessed the activated Caspase-3 (Cas-3) activities in the mesothoracic and metathoracic ganglia to measure CNS programmed cell death after N_2_ anoxia. The ganglionic Cas-3 activity was significantly affected by pretreatments (one-way ANOVA, Control: *n* = 14, Anoxic Control: *n* = 16, Glucose: *n* = 18, 2DG: *n* = 17, MIA: *n* = 16; *F* = 5.945; *p* < 0.001; Fig. 7*C*); glucose increased Cas-3 activity as measured by substrate cleavage, compared with control and 2DG groups (Holm–Sidak multiple comparison; *p* = 0.004 and *p* = 0.016, respectively), MIA similarly exhibited a higher AMC cleavage rate than control and 2DG groups (*p* = 0.005 and *p* = 0.018, respectively). Pretreatment with 2DG had no effect on ganglionic Cas-3 activity compared with control and anoxic control (*p* = 0.76 and *p* = 0.66, respectively), 25 min N_2_ anoxia also had limited effect on Cas-3 activity compared with control (*p* = 0.36).

## Discussion

### SD onset is independent of glycolytic capacity

Pharmacological manipulations of locust CNS glycolytic capacity did not affect parameters of anoxic SD onset in this study, consistent with the theory that insect SD constitutes a secondary adaptation to conserve CNS energy (Robertson et al., 2020). Indeed, delaying anoxic coma onset by reducing cGMP-dependent protein kinase (PKG) activity in *Drosophila* significantly worsens survival and recovery (Dawson-Scully et al., 2010). Additionally, SD induction in locusts is modulated by metabolic stress sensors like NO and AMPK signaling pathways (Armstrong et al., 2009; Rodgers-Garlick et al., 2011; Money et al., 2014), which may act to suppress anaerobic metabolism under anoxia. It must be noted that locusts would not encounter pure N_2_ in their natural habitats, despite the experimental convenience of N_2_-anoxia. Nonetheless, the effects of N_2_-induced SD are similar to that of water submersion, an ecologically relevant hazard for many insects (Robertson and Van Dusen, 2021).

### Discrepancies between 2DG and MIA

The contrasting effects of 2DG and MIA, which are both glycolytic inhibitors, on recovery kinetics and tissue damage patterns are challenging to interpret. Nonetheless, growing evidence suggests that MIA has effects beyond glycolysis inhibition. Iodoacetate derivatives, including MIA, nonspecifically modify a wide range of cysteine containing proteins, including the glycolytic enzyme glyceraldehyde 3-phosphate dehydrogenase (GAPDH; Carneiro et al., 2011). The thiol-oxidizing properties of MIA also contribute to the depletion of cellular glutathione and antioxidant potentials (Schmidt and Dringen, 2009). Notably, the oxidative off-target effects of MIA might have played a significant role in preventing TPP recovery, as MIA inhibition of methionine-sulfoxide reductase (MSR) functions could delay insect anoxic SD recovery and worsen tissue injury (Moskovitz et al., 2000; Franklin et al., 2013; Gu et al., 2016; Suthakaran et al., 2021). It is also worth mentioning the persistent post-SD negativity (which normally should be a transient positivity, see control, glucose, and 2DG groups; Fig. 5), that may suggest MIA challenges locust CNS in ways other than glycolytic inhibition. On the other hand, reports of 2DG’s off-target effects are sporadic and mainly in the context of cell proliferation (Mireuta et al., 2011; Pang et al., 2015).

### Potential mechanism of glycolysis-related damage

Whereas glucose and 2DG have similar effects on recovery kinetics, the underlying mechanisms are likely different. It is possible that glucose hastens recovery by driving aerobic glycolysis and indirectly stimulating oxidative phosphorylation (OXPHOS), evident by the increased CO_2_ output early on during reoxygenation. Such an overall increase in metabolic flux could rapidly replenish neural energy supply and bolster ion clearance capacity, yet it also precipitates latent tissue damage.

We speculate that heightened glycolysis likely contributes to neural injuries by disrupting mitochondrial redox balance and driving ROS production, thereby exacerbating tissue oxidative stress and infarction. During reoxygenation, mitochondria rapidly oxidize glycolytic end products, including lactate, pyruvate, and NADH, generated under both aerobic and anaerobic conditions (Hochachka, 1993). Therefore, high glycolytic flux stimulated by glucose may create a mismatch between substrate supply and mitochondrial demand, fostering conditions for reverse electron transfer (RET) at Complex I (Hoffman and Brookes, 2009).

For instance, Complex I reduces ubiquinone to ubiquinol with electrons from NADH. However, high NADH/NAD^+^ and ubiquinol/ubiquinone ratio, resulting from oversupply of glycolytic end products, make the reverse reaction thermodynamically favorable (Onukwufor et al., 2019). Additionally, the TCA cycle converts accumulated lactate and pyruvate into succinate, which, on subsequent oxidation at Complex II, further increases the level of reduced ubiquinol contributing to RET (Bundgaard et al., 2019). Indeed, the greater CO_2_ output in the glucose group is suggestive of the heightened TCA cycle flux during reoxygenation.

Consequently, the reversal of electron flow favors the formation of superoxide (O_2_•^-^) and reactive oxygen species (ROS) that damages macromolecules and cellular components. These effects potentially give rise to the increased protein carbonyl content observed in glucose group, though it remains unclear why lipid peroxidation decreased simultaneously. Finally, ROS bursts increase the probability of mitochondrial permeability transition that precipitates programed neural cell death, reflected as greater Cas-3 activation in glucose group.

### Potential mechanisms underlying 2DG’s effects

Given that the glycolytic inhibitor 2DG also expedited the recovery of TPP and neuronal excitability similar to glucose, we speculate 2DG-animals may have switched to β-oxidation to fuel recovery (McMullen et al., 2023). Fatty acids metabolism is slower but generates greater energy compared with glycolysis, which may explain the initial low CO_2_ output in 2DG group. Although insect CNS stores little lipids, glial cells may mobilize peripheral fat body reserves when glycolysis is impaired, and supply ketone bodies to neurons as oxidative fuel (Rittschof and Schirmeier, 2018). For instance, it is thought that *Drosophila* glia secretes Glaz (homologous to apolipoprotein ApoD) to communicate with the fat body during starvation (McMullen et al., 2023); a similar mechanism likely exists in the locust CNS, considering the central role of fatty acids in powering flight muscles (Haunerland, 1997). In the context of anoxic SD recovery, glycolysis block coupled with increased β oxidation may help prevent the oversupply of TCA cycle substrates and preserve the stoichiometric balance of intermediates. As such, the probability of RET and ROS production during reoxygenation would likely decrease, agreeing with the trend of reduced protein carbonylation in 2DG group.

A peculiar effect of 2DG is the increased magnitude of the post-SD positivity. It is worth noting that a similar form of DC positivity is observed following seizures, SD, and periods of heightened neuronal activities in a number of mammalian studies (Gutnick et al., 1979; Haglund and Schwartzkroin, 1990; Kawasaki et al., 1990; D’Ambrosio et al., 2002).

There are two potential mechanisms that could underlie the origin of this positive shift of TPP following recovery. First, the DC positivity may be the consequence of higher Na^+^/K^+^ ATPase activity during SD recovery. The loss of ion homeostasis during SD could trigger an increase in pumping rates to restore ion gradients. However, the heightened pump activity may persist even after the restoration of physiological level of [K^+^]_o_, leading to an undershoot that is reflected as a slight positive shift of TPP. The subsequent return of [K^+^]_o_ and TPP to the preanoxic baseline level is thought to be mediated by glial K^+^ extrusion mechanisms involving KIR channels (D’Ambrosio et al., 2002). Given the glycolytic preference of insect glia (Rittschof and Schirmeier, 2018), 2DG may interfere with these processes, resulting in greater post-SD positivity.

An alternative explanation of the DC positivity is the differential recovery rates of the component membrane potentials of TPP. The insect TPP represents the potential difference between two polarities of the barrier-forming perineurial cell layer; commonly denoted as the basolateral potential (V_b_, intracellular vs hemolymph) and the adglial potential (V_a_, intracellular vs CNS interstitium, see Extended Data Fig. 5-1; Schofield and Treherne, 1984; Robertson et al., 2020). Accordingly, TPP recovery trajectory is dependent on the opposing exponential recoveries of V_a_ and V_b_ during reoxygenation (with time constants τ_a_ and τ_b_). Importantly, when V_a_ recovers faster than V_b_, the TPP develops a transient positive shift that diminishes after both V_a_ and V_b_ returns to preanoxic baseline level (Extended Data Fig. 5-1). It is likely that the effects of 2DG are primarily driven by accelerated V_a_ recovery and reduced τ_a_, resulting in a faster TPP recovery rate and greater post-SD positivity. 2DG may shorten τ_a_ by inhibiting uncontrolled excitations, which conserves energy and accelerates the return of CNS ion homeostasis. This effect may also explain the faster re-establishment of ventilatory motor patterning from un-patterned excitations in 2DG group.

Incidentally, 2DG’s anticonvulsant effects are well-documented (Wijayasinghe et al., 2022; Sutula and Fountain, 2023), although it is unclear whether the insect neuromuscular hyperexcitability during SD onset and recovery is analogous to mammalian epileptiform bursts. In any case, the mechanism behind 2DG’s impact on recovery trajectory warrants further investigation.

### Merit of low metabolism during reoxygenation

The CNS is particularly vulnerable to anoxic damage in two major ways: (1) energy depletion during anoxia leads to necrotic cell death and subsequent apoptosis (Callizot et al., 2019; Lemale et al., 2022; Tuo et al., 2022), and (2) reactivation of the stalled electron transport chain (ETC) during reoxygenation causes oxidative damage, worsening tissue injury (Wu et al., 2020; Jaganjac et al., 2022). In the current experiments, supplying a metabolic fuel source (glucose) and an inhibitor (2DG) both improved recovery rates, but only glucose increased tissue damage and CO_2_ output during early reoxygenation. Given such observations, we propose that acute glycolysis block by 2DG during anoxic SD recovery could be neuroprotective, and that high glycolytic flux and OXPHOS activity during reoxygenation may contribute to tissue injury in the insect CNS.

The protective effects of 2DG and carbohydrate restriction in the context of energy stress are not novel; however, as far as we are aware, our results represent the first documented acutely induced neuroprotection by this compound during anoxic SD 2DG has long been used to mimic the effect of dietary restriction on ischemic stroke and neurodegenerative diseases (Yu and Mattson, 1999; Pajak et al., 2019). Nonetheless, past studies often employed treatment periods lasting from days to weeks, that incur global changes in metabolic and gene expression profiles. In contrast, the effects observed in our study are likely to be induced by glycolysis inhibition alone, given the brief pretreatment periods (20–30 min) and high dose (50 mm or 820 mg/dl).

Given that the return of neural functioning precedes muscle activation, the first 20 min of whole-animal CO_2_ production likely reflects the pattern of CNS metabolism well. Therefore, it is possible that blocking glycolysis may allow a slower and perhaps “smoother” reactivation of mitochondrial ETC during reoxygenation, through modulating substrate availability and thus the thermodynamic driving forces of respiration. Whether this was achieved through glycolysis inhibition alone or involves glial β oxidation requires further verification. Indeed, the ability to down-regulate glycolysis during oxygen reperfusion constitutes an important molecular response to aid the turtle’s survival during anoxia (Bundgaard et al., 2019). As noted above, the elevated oxidative damage and cell death in glucose-treated animals are likely because of the greater respiratory activity than tissue demands, which counterintuitively jeopardizes animal survival despite providing a temporary energy boost during reoxygenation.

### Limitations

A major limitation of the current results is the discrepancies between the effects of 2DG and MIA, which requires further experimentation to resolve. Despite potential off-target effects, iodoacetates are historically reliable glycolytic inhibitors (Fuhrman and Field, 1943; Cano‐Ramírez et al., 2012). We cannot exclude the possibility that MIA’s effect mainly arises from GAPDH inhibition, or indeed if 2DG exhibited unknown off-target effects. Additionally, it is essential to note that our experiments do not account for necrotic cell death during anoxia. It is entirely possible that anaerobic glycolysis reduced uncontrolled cell lysis in the depolarized state by maintaining a small degree of ion homeostasis (Campbell et al., 2018). Indeed, we observed an attenuated SD amplitude in the glucose group only. As the negative shift of TPP during SD corresponds well with the surge of [K^+^]_o_ (Robertson et al., 2020), a lower SD amplitude is suggestive of reduced [K^+^]_o_, potentially facilitated by anaerobic ion pumping (Armstrong et al., 2012). Future experiments should directly compare how glycolytic capacity affect neural necrosis during anoxia.

### Conclusion and prospects

In summary, we present evidence that glycolysis is not a critical metabolic component in either anoxic SD onset or recovery in the locust CNS; we further propose the detrimental role of heightened glycolysis during reoxygenation that may lead to oxidative damage. Correspondingly, acute glycolysis inhibition through 2DG hastens SD recovery and potentially protects against anoxia-reoxygenation damage. Nonetheless, the precise physiological effects elicited by glycolytic inhibition at the cellular level remain unclear and warrant further investigation. Moreover, it will be useful to assess how animal glycolytic capacity affects the long-term behavioral outcomes and fitness following anoxia.

Elucidating how metabolic flux is controlled during the critical period of anoxia-reoxygenation is necessary to improve our understanding of strategies and mechanisms of insect anoxia tolerance. Also, similar experiments could be performed with mammalian ischemic SD models. Considering the prolific use of 2DG and its derivatives in human imaging studies, its effect on CNS glycolysis may have clinical significance in improving neurologic outcomes following stroke or cerebral ischemia.

## Synthesis

Reviewing Editor: Eran Perlson, Tel Aviv University Decisions are customarily a result of the Reviewing Editor and the peer reviewers coming together and discussing their recommendations until a consensus is reached. When revisions are invited, a fact-based synthesis statement explaining their decision and outlining what is needed to prepare a revision will be listed below. The following reviewer(s) agreed to reveal their identity: NONE. Note: If this manuscript was transferred from JNeurosci and a decision was made to accept the manuscript without peer review, a brief statement to this effect will instead be what is listed below.

The auteurs answer reviewers concerns, and the manuscript is ready for publication

## Author Response

Reviewer #1 Review

Rationale for Significance Rating for Authors (Required):

There are clearly important differences between insects and humans. In particular, the roles of transmitter systems can be strikingly different. It is all the more interesting that basic features of spreading depolarizations are so well comparable between locusts and humans. The effects of 2-DG glucose are very interesting in the present work and quite inspiring beyond the field of entomologists.

Thank you for this positive assessment.

Assessment of the Manuscript for Authors (Required):

In principle, this is an interesting study. The authors investigated the effects of varying glycolytic capacity on adult female locust anoxic SD parameters, using glucose or the glycolytic inhibitors 2-deoxy-D-glucose (2DG) or monosodium iodoacetate (MIA). One problem is that the findings of 2-DG and MIA do not match at all, even though both substances inhibit glycolysis. It would be more transparent to state this problem in the abstract.

We have updated the abstract with a description of this issue.

On the other hand, glucose and 2-DG have partially similar effects. I would also point out in the abstract that the authors have understood that these findings are not easy to interpret.

We have updated the abstract to call for cautious interpretation of our results.

However, the experiments make a sound impression and it is typical of honest scientific work that not everything can be resolved and explained. Again, the findings are interesting, the discussion provides a good basis to contemplate the results.

A main point is that the “overshoot of the TPP” very likely corresponds to the potassium undershoot that is usually recorded after SD, seizures and high-frequency stimulation (note that the reference electrode in mammalian in vivo recordings is also at the outer side of the BBB; so your set-up is quite similar in this respect as far as I understand it). In mammals, this potential change has been related by different groups to overactivation of the sodium-potassium pump, e.g.(D’Ambrosio et al., 2002; Gutnick et al., 1979; Haglund and Schwartzkroin, 1990; Heinemann and Lux, 1975; Kawasaki et al., 1990) In mammals, the potassium undershoot is accompanied by a corresponding post-SD positivity in the DC potential. This most likely origin of the potential change should be discussed, as exactly the same pattern seems to be observed in SD in mammals. In mammals, this “overshoot” typically becomes smaller with metabolic challenge of the tissue which fits with your findings.

We have updated the discussion to include this potential mechanism for the overshoot or post-SD positivity. See section “Potential mechanisms underlying 2DG’s effects”. It is truly thought-provoking that the DC positivity is also widely observed in mammals. We agree that increased NKA activation may account for the positivity. However, with re-examination of our previous [K+]o measurements, we cannot positively identify whether or not a K+ undershoot accompanies the post-SD positivity. Nonetheless, this possibility should still be investigated in future studies.

Another related point is yet that the term “overshoot” is dangerous. In the clinics, DC traces are oriented with negativity upward and positivity downward according to the traditional polarity rule of electroencephalography (EEG) (Ham, 2018). Thus, a clinician would call your “TPP overshoot” a “TPP undershoot”. There are in fact very good reasons for the EEG polarity rule, which are beyond the scope of this review. In any case, either you follow the EEG polarity rule and change all your figures (negativity upward, positivity downward) or you should simply call your “TPP overshoot” “post-SD positivity”. Otherwise, this will be yet another source of confusion in the field.

We appreciate the input, the terminology has been updated to “post-SD positivity”.

“Some studies, for instance, show that hyperglycemia and anaerobic glycolysis enhance tissue SD resistance and reduce injury under cerebral ischemia

(DiNuzzo et al., 2012; Hartings et al., 2008; Hertz et al., 2015; Hoffmann et al., 2012; Wotton et al., 2020), while others associate excess glucose supply and neuronal glycolysis with worsened secondary damage post-SD (Barros et al., 2020; De Courten-Myers et al., 1994; Herrero-Mendez et al., 2009, 2009; Li et al., 2018).” It should be mentioned in this context that the dependence of ischemic cell death on serum glucose presumably describes a U-shaped curve in mammals with brain injuries.(Dreier and Reiffurth, 2015) Hyperglycemia seems bad, but so does relative hypoglycemia. For example, Eagles et al. wrote in their recent clinical study on aneurysmal subarachnoid hemorrhage (aSAH)(Eagles et al., 2022): “Our work identified a potential target for glucose levels after aSAH because individuals who maintained levels below 9.2 mmol/L (∼166 mg/dl) had a significant decrease in their risk of unfavorable outcomes at 3 months. It should also be noted that, although 9.2 mmol/L is lower than the common threshold for hyperglycemia, it is higher than many of the previously investigated aggressive glucose targets in neurocritical care,13 which may partially explain our significant result.”

We have updated the introduction to include this information as suggested, see the end of the second paragraph.

“Thus, while sometimes fully recoverable and benign, SD frequently predisposes neural tissues to injury and cell death due to energy depletion; in the case of anoxic SD, such injury can be further compounded by oxygen reperfusion damage (Gomez et al., 2022).”

The sentence is somehow correct, but I think it should be added that SD is the characteristic change in the state of neurons that practically always immediately precedes death, at least in higher animals. I.e. the dying process in mammals is inseparable from the SD process with few exceptions.(Oliveira-Ferreira et al., 2020) Of course, this does not mean that SD is always irreversible but it can become irreversible under certain conditions. For example, I would write something like: “Thus, while often fully recoverable and relatively benign, SD predisposes neural tissues to injury and cell death. At least in higher animals, including humans, SD is the characteristic change in the state of neurons that virtually always occurs in the dying process, i.e., at the transition from life to death.(Carlson et al., 2019; Dreier et al., 2018; Dreier et al., 2019)”

We have updated the introduction to incorporate this suggestion, see the end of the first paragraph.

I would be cautious about reperfusion injury. In patients, it is now crystal clear that neurons without reperfusion die, but some or even all neurons with reperfusion survive. Otherwise, all the clinical studies about intravenous thrombolysis with rt-PA or mechanical recanalization would have been negative. In the early time window, reperfusion is the only effective treatment for ischemic stroke, and in many cases a highly effective one. The same applies to cardiac arrest and resuscitation of course.

We have modified the introduction to incorporate this feedback, see the end of the first paragraph. We originally meant to underline that the process of reperfusion itself can introduce additional stress, which is a unique challenge to hypoxic/anoxic SD. The wording might have been a source of confusion.

Minor points are the following:

“Such a process involves large surges of extracellular potassium ([K+]o), intracellular sodium and calcium ([Na+]I, [Ca2+]i, respectively)”

I think that the bracket should not be after “respectively”, but after “[Ca2+]i”.

Updated according to suggestion.

“Adult Locusta migratoria aged 4 - 5 weeks past adult molt...” It should be “moult” rather than “molt”.

Updated according to suggestion.

“The final concentrations of the drugs used in all experiments were: 10 mM for glucose,...” In the clinic, glucose is often given in mg/dl. Maybe, the authors could write “10 mM (180 mg/dl)...” to ease understanding by clinicians used to mg/dl. What is the glucose reference range for locusts? The normal range should be mentioned in the Methods.

Updated according to suggestion. Included the reference range of glucose during and after feeding in methods, note that trehalose is the major blood sugar in insects instead of glucose.

“2DG-animals may have switched to glial cell beta-oxidation to fuel recovery (McMullen et al., 2023). Fatty acids metabolism is slower but generates greater energy compared to glycolysis, which may explain the initial low CO2 output in 2DG group. In addition to promoting beta oxidation, glycolytic inhibition may have helped conserve cellular energy to aid recovery, evident by the increased the post-anoxic TPP overshoot in 2DG group.” Grammar should be improved.

This content has been modified according to the other reviewer’s suggestion.

Reviewer #2 Review

Rationale for Significance Rating for Authors (Required):

The effect of glycolysis in locust has not been studied much and thus could be of interest to people in this field. However, the results at its current stage are mostly descriptive.

We appreciate this feedback and have updated the discussion to include more mechanistic analysis of the results (see below).

Assessment of the Manuscript for Authors (Required):

The effect of glycolysis on anoxic spreading depolarization (SD) and recovery is examined in locust.

SD onset was not affected by either glucose and 2DG. However, they expedited the recovery of CNS electrical activity during reoxygenation.

The team measured the protein carbonyls levels and tissue malondialdehyde (MDA) content to quantify protein oxidation and membrane lipid peroxidation. The result showed that glucose and MIA, but not 2DG, increased oxidation which suggests tissue damage. The main conclusion is that glycolysis is not a critical metabolic component in either anoxic SD onset or recovery, and that heightened glycolysis during reoxygenation exacerbates CNS injuries.

The result is of interest to understand the effect of glycosis and SD in locust, which has not been examined extensively. The experiment design is clear and straightforward. The data are clearly presented and easy to follow. However, The result is most descriptive regarding the effect of three drugs, 2DG glucose and MIA. There is not much information regarding the mechanism.

The discussion has been restructured to emphasize the potential mechanisms supported by our observations, particularly in terms of glucose-related damage, and various effects of 2DG. Some of the potential mechanisms involved and the physiological effects of 2DG are still areas of active research, we hope that our results can provide some ideas for the future studies to come.

Response to the Editor

We appreciate the feedback regarding the ms. We have updated the wordings in the abstract and discussion to be less decisive with our conclusions. In an effort to increase transparency, we have also included a “limitation” section outlining the challenges of interpreting the discrepancies found in our results. Lastly, we made significant modifications to the discussion section, in order to improve the mechanistic understanding of our observations.
